# Supplementary material for: An Investigation on Glucuronidation Metabolite Identification, Isozyme Contribution, and Species Differences of GL-V9 In Vitro and In Vivo
Source: Molecules. 2019 Apr 22;24(8):1576. doi: 10.3390/molecules24081576 (PMC6515479; doi:10.3390/molecules24081576)
Supplement: Supplementary file 1 [file molecules-24-01576-s001.pdf]

The pharmacokinetic parameters of GL-V9 in rats (n=6, mean ± SD)

| Parameter | Units | oral administration (50 mg/kg) |
|---|---|---|
| $T_{1/2}$ | h | 3.08 ± 1.05 |
| $T_{max}$ | h | 0.31–0.77 |
| $C_{max}$ | ng/mL | 167.33 ± 50.05 |
| $AUC_{(0-t)}$ | h*ng/mL | 1212.06 ± 246.47 |
| $AUC_{(0-inf)}$ | h*ng/mL | 1256.04 ± 271.82 |
| $V_d$ | L/kg | 181.06 ± 63.57 |
| $MRT_{0-t}$ | h | 7.70 ± 0.91 |
| CL | L/h/kg | 41.38 ± 8.84 |
| F | - | 8.54% |

* These data cited from the Xing et al. [38].
